# Network Pharmacology and Molecular Docking Identify Medicarpin as a Potent CASP3 and ESR1 Binder Driving Apoptotic and Hormone-Dependent Anticancer Activity

**DOI:** 10.3390/ijms27010174

**Published:** 2025-12-23

**Authors:** Yanisa Rattanapan, Sirinya Sitthirak, Aman Tedasen, Thitinat Duangchan, Hasaya Dokduang, Nawanwat C. Pattaranggoon, Krittamate Saisuwan, Takol Chareonsirisuthigul

**Affiliations:** 1 Department of Medical Technology, School of Allied Health Sciences, Walailak University, Nakhon Si Thammarat 80160, Thailand; yanisa.rt@wu.ac.th (Y.R.); sirinya.sit@wu.ac.th (S.S.); aman.te@wu.ac.th (A.T.); thitinat.du@wu.ac.th (T.D.); 2 Hematology and Transfusion Science Research Center (HTSRC), Walailak University, Nakhon Si Thammarat 80160, Thailand; 3 Research Excellence Center for Innovation and Health Products (RECIHP), Walailak University, Nakhon Si Thammarat 80160, Thailand; 4 Faculty of Medicine, Mahasarakham University, Mahasarakham 44000, Thailand; hasaya.d@msu.ac.th; 5 Cholangiocarcinoma Research Institute, Khon Kaen University, Khon Kaen 40002, Thailand; 6 Faculty of Medical Technology, Rangsit University, Muang 12000, Thailand; nawanwat.p@rsu.ac.th; 7 Department of Immunopharmacology, Graduate School of Medicine, Kyoto University, Kyoto 606-8507, Japan; saisuwan.krittamate.2y@kyoto-u.ac.jp; 8 Department of Pathology, Faculty of Medicine Ramathibodi Hospital, Mahidol University, Bangkok 10400, Thailand

**Keywords:** medicarpin, ovarian cancer, network pharmacology, molecular docking, PI3K-Akt/mTOR pathway, ESR1, CASP3

## Abstract

Ovarian cancer (OC) remains one of the most lethal gynecologic malignancies due to late diagnosis, rapid progression, and frequent chemoresistance. Despite advances in targeted therapy, durable responses are uncommon, underscoring the need for novel multitarget agents capable of modulating key oncogenic networks. Medicarpin, a natural pterocarpan phytoalexin, exhibits diverse pharmacological activities; however, its molecular mechanisms in OC are poorly defined. This study employed an integrative in silico framework combining network pharmacology, pathway enrichment, molecular docking, and survival analysis to elucidate medicarpin’s therapeutic landscape in OC. A total of 107 overlapping targets were identified, resulting in a dense protein–protein interaction network enriched in kinase-mediated and apoptotic signaling pathways. Ten hub genes were emphasized: CASP3, ESR1, mTOR, PIK3CA, CCND1, GSK3B, CDK4, PARP1, CHEK1, and ABL1. Gene Ontology and KEGG analyses demonstrated substantial enrichment in the PI3K–Akt/mTOR and prolactin signaling pathways. Docking revealed the stable binding of medicarpin to CASP3 (−6.13 kcal/mol) and ESR1 (−7.68 kcal/mol), supporting its dual regulation of hormonal and apoptotic processes. Although CASP3 and ESR1 expression alone lacked prognostic significance, their network interplay suggests synergistic relevance. Medicarpin exhibits multitarget anticancer potential in OC by modulating kinase-driven and hormone-dependent pathways, warranting further experimental validation.

## 1. Introduction

Ovarian cancer (OC) continues to be one of the most fatal gynecological cancers globally, with elevated morbidity and mortality mainly due to late-stage detection, rapid disease advancement, and the emergence of chemoresistance. Despite advancements in surgical techniques and systemic therapies, including platinum-based chemotherapy, poly (ADP-ribose) polymerase (PARP) inhibitors, and anti-angiogenic agents, the five-year survival rate for individuals with advanced ovarian cancer remains dismal [1,2,3]. This highlights the pressing need for novel therapeutic approaches that can enhance patient outcomes and overcome resistance mechanisms.

The pathobiology of OC is marked by significant molecular heterogeneity and the deregulation of many oncogenic signaling pathways, such as PI3K/AKT/mTOR, MAPK, NF-κB, and apoptosis-related networks [4,5]. Conventional single-target drug discovery has often proven insufficient in tackling intricate molecular interactions. In this context, natural compounds have emerged as a viable source of anticancer medicines, providing multitarget efficacy, chemical diversity, and relatively favorable safety profiles [6,7,8].

**Figure 1:**
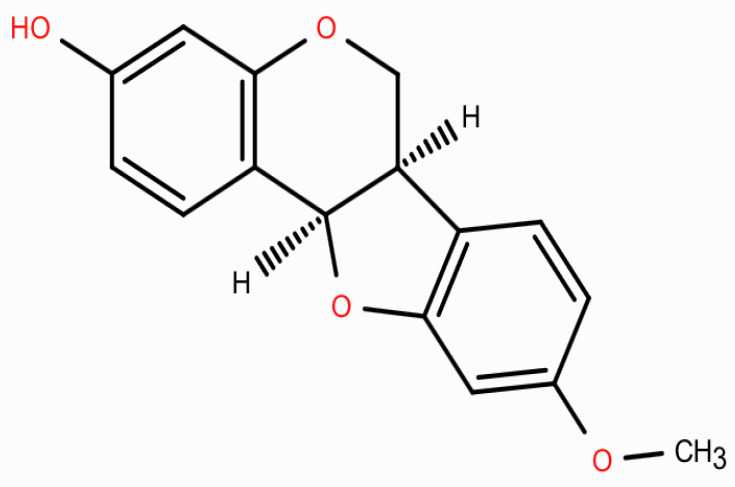
The two-dimension chemical structure of medicarpin. The red color indicates heteroatoms (oxygen atoms) in the chemical structure of medicarpin, highlighting functional groups such as hydroxyl and ether moieties.

Medicarpin, a naturally occurring pterocarpan phytoalexin predominantly derived from Dalbergia species, demonstrates various pharmacological activities, including anti-inflammatory, antioxidant, osteogenic, and anticancer effects, through the modulation of NF-κB, MAPK, and apoptosis-related pathways, highlighting its potential as a promising small-molecule candidate [9,10,11]. The chemical structures, depicted in two-dimensional formats, are provided in Figure 1. Preclinical investigations have demonstrated its capacity to induce apoptosis, inhibit tumor growth, regulate oxidative stress, and disrupt PI3K/AKT and MAPK pathways in diverse cancer models [12,13]. Nonetheless, its therapeutic potential and molecular targets in OC remain predominantly unexamined.

Network pharmacology, a nascent field that combines computational biology, chemoinformatics, and systems pharmacology, offers a robust framework to clarify the polypharmacological characteristics of natural chemicals [14,15,16]. Network pharmacology can elucidate mechanistic insights into therapeutic action within the intricate molecular landscape of cancer by mapping compound-target-disease connections and performing route enrichment analysis. Additionally, molecular docking provides structural confirmation by forecasting the binding affinity and stability of candidate drugs with essential oncogenic proteins [17]. Collectively, these computational approaches provide a logical prioritization of innovative treatment options before experimental validation.

Although there are encouraging signs, the precise mechanisms through which medicarpin influences oncogenic signaling and apoptosis-related pathways in OC have yet to be investigated. No comprehensive investigations have yet combined network pharmacology with molecular docking to elucidate its primary targets and pathways in this cancer.

The current study sought to identify the potential molecular targets and regulatory mechanisms of medicarpin in OC using an integrative in silico approach that includes target prediction, protein–protein interaction (PPI) network analysis, enrichment analysis, and molecular docking validation. This framework aims to offer mechanistic insights and establish a basis for future experimental and translational research on medicarpin as a potential therapeutic candidate for OC.

## 2. Results

### 2.1. Workflow of Integrative Network Pharmacology and Molecular Docking Analysis for Medicarpin in Ovarian Cancer

**Figure 2:**
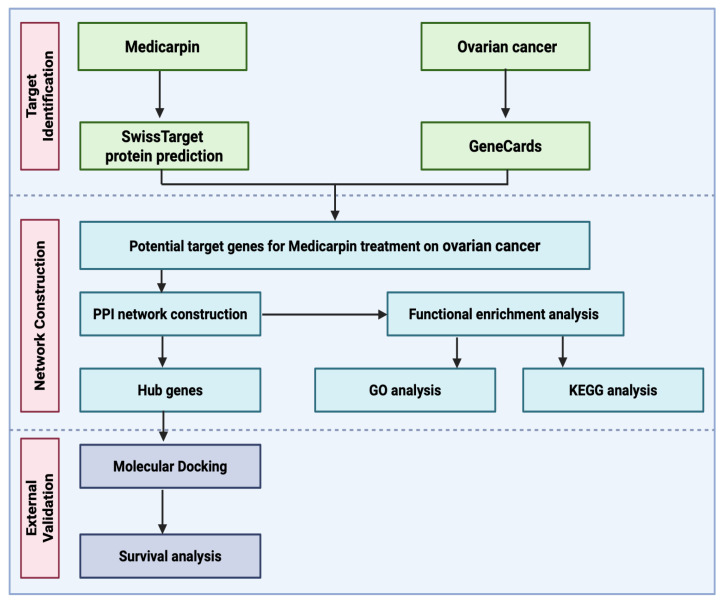
Overview of the network pharmacology and molecular docking methodologies employed in the present study.

The comprehensive workflow employed in this study is illustrated in Figure 2. Potential medicarpin targets were initially identified using the SwissTargetPrediction database, whereas OC-related genes were obtained from the GeneCards database. Genes that interact between medicarpin-related and OC-associated targets were identified as potential therapeutic targets. The overlapping targets were then analyzed for network creation and functional characterization. A protein–protein interaction (PPI) network was constructed to identify hub genes, followed by functional enrichment analyses using Gene Ontology (GO) and Kyoto Encyclopedia of Genes and Genomes (KEGG) pathways to clarify the biological functions and signaling pathways involved. Molecular docking was subsequently conducted to confirm the anticipated interactions between medicarpin and the hub proteins. Ultimately, survival analysis was performed to evaluate the prognostic relevance of critical target genes in OC patients.

### 2.2. Physicochemical Properties and ADMET Characterization of Medicarpin

The physicochemical and pharmacokinetic characteristics of medicarpin were methodically assessed utilizing the SWISS, pKCSM, and ProTox-III servers to determine its viability as a drug-like entity (Supplementary Tables S1–S3). Medicarpin (C_16_H_14_O_4_) has a molecular weight of 270.28 g/mol and a topological polar surface area (TPSA) of 47.92 Å^2^, both of which fall within the ideal range for oral bioavailability. The molecule comprised one hydrogen-bond donor and four acceptors, with a minimal count of rotatable bonds (n = 1), signifying structural stiffness that facilitates stable receptor attachment. The computed consensus Log P = 2.53 indicates moderate lipophilicity, which enhances membrane permeability while preserving water solubility. Medicarpin was anticipated to exhibit solubility ranging from soluble to moderately soluble across all three solubility models (ESOL, Ali, SILICOS-IT), indicating its suitability for formulation without the need for solubility-enhancing changes.

The assessment of drug-likeness established that medicarpin meets all five criteria of Lipinski, Ghose, Veber, Egan, and Muegge, with no alerts from PAINS or Brenk, suggesting a slight risk of assay interference or reactive liabilities. A bioavailability score of 0.55 and a synthetic accessibility of 3.54 indicate intermediate synthetic feasibility and favorable absorption potential. Medicarpin exhibited significant gastrointestinal absorption and permeability across the blood–brain barrier, suggesting its potential to traverse physiological barriers pertinent to both peripheral and central pharmaceutical targets.

The pKCSM-based ADMET modeling corroborated these findings. Medicarpin exhibited significant intestinal absorption (95.19%) and considerable Caco-2 permeability (log Papp = 1.246), aligning with its physicochemical equilibrium between lipophilicity and polarity. It was not a substrate for P-glycoprotein, indicating a negligible risk of efflux-mediated loss of bioavailability. The chemical demonstrated a moderate volume of distribution (log VDss = 0.065) and a low unbound percentage (Fu = 0.04), signifying persistent plasma binding. Medicarpin was anticipated to traverse the blood–brain barrier (log BB = 0.324) but exhibited low central nervous system permeability (log PS = −1.838), indicating restricted central accumulation.

Medicarpin was identified in metabolic prediction models as a substrate of CYP3A4 and a potential inhibitor of CYP1A2, CYP2C9, CYP2C19, CYP2D6, and CYP3A4, indicating its capacity to affect multiple cytochrome P450 isoforms. While this may increase the likelihood of drug–drug interactions, such multitarget modulation could also contribute to its pharmacological plasticity. The overall clearance rate of 0.273 mL/min/kg and the non-substrate designation for OCT2 suggest advantageous elimination kinetics.

Toxicological assessments revealed no hepatotoxicity, no hERG inhibition, and no skin sensitization, suggesting minimal cardiotoxic and dermatologic risk. The oral rat acute toxicity (LD_50_ = 2.512 mol/kg) and chronic toxicity (LOAEL = 1.875 log mg/kg/day) metrics indicate a moderate safety profile, whereas the AMES positive implies possible mutagenesis signals that necessitate experimental validation.

ProTox-III modeling categorized medicarpin as active for neurotoxicity (probability = 0.87) and respiratory toxicity (0.98), while indicating inactivity for nephrotoxicity and cardiotoxicity. Medicarpin exhibited activity towards aromatase and estrogen receptor α (ER-α and ER-LBD) within nuclear receptor signaling pathways, aligning with its phytoestrogenic structure, while demonstrating inactivity in androgen, PPAR-γ, and AhR pathways. It showed immunotoxic activity (0.96), but it was not carcinogenic or mutagenic, which suggests that it modulates the immune system in a selective way rather than being genotoxic.

These computational findings collectively underscore medicarpin as a drug-like natural molecule characterized by favorable oral bioavailability, moderate lipophilicity, metabolic flexibility, and minimal systemic toxicity. The anticipated interactions with ER-α and CYP-related enzymes correspond with its potential multitarget mechanism in hormone-responsive and signaling-driven malignancies, including OC.

### 2.3. Identification of Potential Therapeutic Targets and Network Construction of Medicarpin in Ovarian Cancer

**Figure 3:**
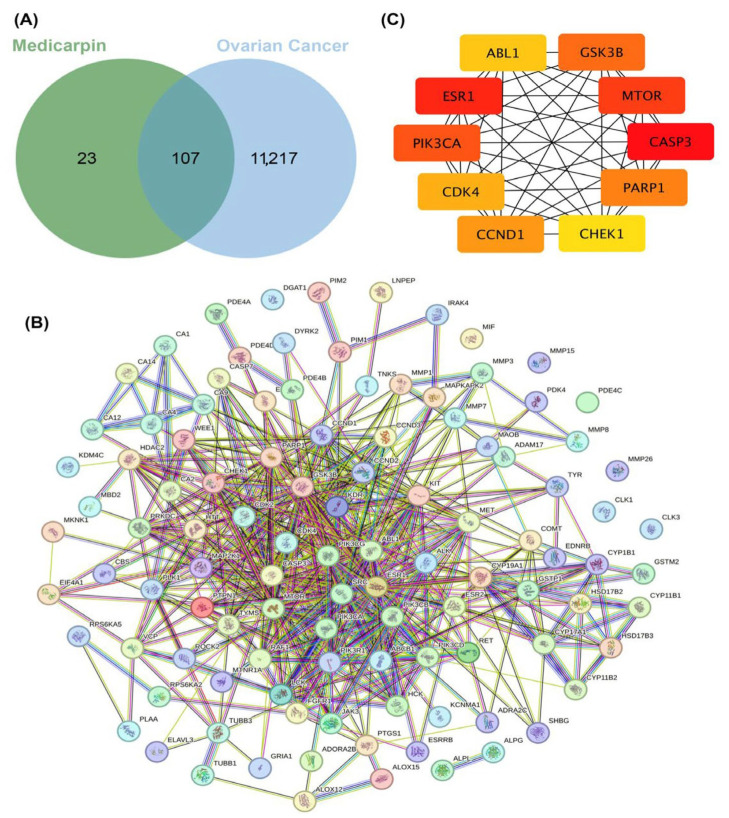
Identification of potential therapeutic targets of medicarpin in ovarian cancer. (**A**) Venn diagram showing 107 overlapping targets between medicarpin and ovarian cancer genes. (**B**) Protein–protein interaction (PPI) network of overlapping targets constructed using STRING. (**C**) Topological analysis identifying ten hub genes (CASP3, ESR1, MTOR, PIK3CA, CCND1, GSK3B, CDK4, PARP1, CHEK1, and ABL1) as key regulatory nodes.

To clarify the molecular mechanisms that contribute to the therapeutic potential of medicarpin in OC, prospective targets were identified using the SwissTargetPrediction database, and OC-related genes were sourced from GeneCards. A total of 130 targets connected with medicarpin and 11,324 genes related to OC were discovered. The intersection analysis identified 107 overlapping genes, deemed potential therapeutic targets of medicarpin for OC (Figure 3A).

A protein–protein interaction (PPI) network was subsequently established utilizing the STRING database to investigate the functional interactions among these targets (Figure 3B). The resultant network exhibited dense linkages, indicating a complex interaction framework through which medicarpin may influence many signaling pathways.

Topological analysis using the cytoHubba plug-in in Cytoscape identified ten hub genes with the highest degree values: CASP3, ESR1, MTOR, PIK3CA, CCND1, GSK3B, CDK4, PARP1, CHEK1, and ABL1 (Figure 3C). These hub genes signify prospective molecular targets for the future confirmation of medicarpin’s anticancer activities in OC.

### 2.4. Gene Ontology Enrichment Reveals Kinase-Mediated and Apoptotic Pathways as Core Mechanisms of Medicarpin Action in Ovarian Cancer

To clarify the molecular mechanisms of medicarpin’s anticancer efficacy in OC, Gene Ontology (GO) enrichment analysis was performed on the 44 common targets identified from the network pharmacology screening. A total of 1842 Gene Ontology (GO) items were substantially enriched (FDR < 0.05), comprising 983 biological processes (BP), 438 cellular components (CC), and 421 molecular functions (MF), illustrating the diverse regulatory impacts of medicarpin on tumor biology.

**Figure 4:**
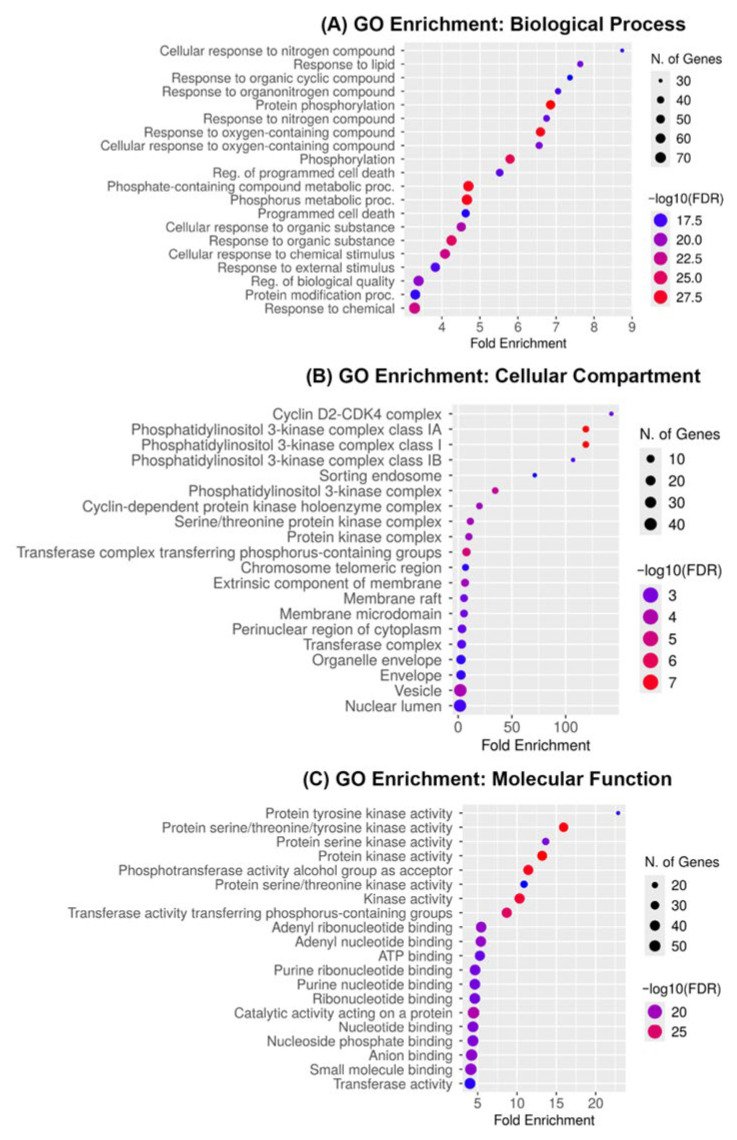
Gene Ontology enrichment analysis of medicarpin targets in ovarian cancer. (**A**) Biological process enrichment showing significant modulation of phosphorylation, apoptosis, and cellular response pathways. (**B**) Cellular component enrichment indicating localization to PI3K and CDK complexes, membrane rafts, and chromosomal regions. (**C**) Molecular function enrichment highlights kinase activity, ATP binding, and nucleotide binding as key molecular features. Bubble size represents the number of genes, and the color scale indicates −log_10_(FDR).

In the biological process ontology (Figure 4A), the most significantly enriched terms included protein phosphorylation, regulation of programmed cell death, phosphorus metabolic activity, and cellular response to chemical and organic chemicals. These enrichments suggest that medicarpin may disrupt signaling pathways governing cell survival, apoptosis, and oxidative stress responses, therefore influencing neoplastic development in OC cells.

In the cellular component category (Figure 4B), the following enriched phrases were found: phosphatidylinositol 3-kinase (PI3K) complex, cyclin-dependent protein kinase complex, membrane raft, and chromosomal region. The data indicate that medicarpin targets are primarily located within membrane-associated and nuclear signaling complexes, which are essential for cell-cycle regulation and intracellular signal transduction.

In the molecular function ontology (Figure 4C), the predominant terms included protein tyrosine kinase activity, serine/threonine kinase activity, ATP binding, and nucleotide binding. The significant prevalence of kinase-related actions highlights kinases as primary pharmacological targets of medicarpin, aligning with its anticipated affinity for crucial regulators, such as mTOR, PI3K, and CDK2.

The GO enrichment analysis indicates that medicarpin predominantly exerts its anticancer effects by modulating kinase-mediated signaling pathways and apoptotic mechanisms in OC. These data corroborate the concept that medicarpin operates as a multi-target small drug, adept at modulating oncogenic phosphorylation networks and facilitating tumor cell apoptosis.

### 2.5. KEGG Pathway Enrichment and Functional Mapping Identify PI3K-Akt and Prolactin Signaling as Central Mechanisms of Medicarpin Action in Ovarian Cancer

To elucidate the signaling cascades responsible for the pharmacological effects of medicarpin in OC, a Kyoto Encyclopedia of Genes and Genomes (KEGG) pathway enrichment analysis was performed on the 44 overlapping targets derived from the network pharmacology dataset. One hundred and fifty-six pathways were significantly enriched (FDR < 0.05), highlighting the considerable biological significance of medicarpin-associated targets.

**Figure 5:**
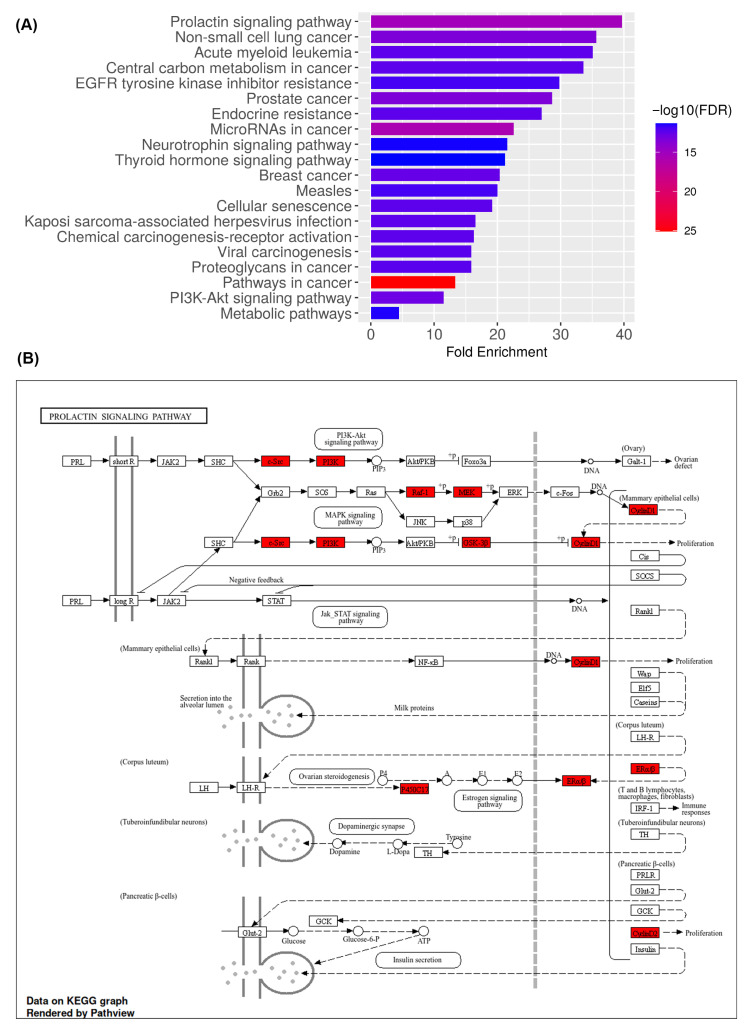
KEGG pathway enrichment and mapping of medicarpin targets in ovarian cancer. (**A**) KEGG enrichment analysis of 44 overlapping targets, highlighting major oncogenic and endocrine-related pathways, including PI3K–Akt and prolactin signaling. Bubble size represents the number of genes, and color intensity indicates statistical significance (−log_10_ FDR). (**B**) Pathview visualization of the prolactin signaling pathway showing medicarpin-associated targets (red) positioned at key regulatory nodes such as PIK3CA, AKT1, MAPK1, ESR1, and SOCS3.

Figure 5A demonstrates that the most enriched pathways are primarily associated with oncogenic and endocrine signaling, encompassing the prolactin signaling pathway, PI3K-Akt signaling pathway, resistance to EGFR tyrosine kinase inhibitors, central carbon metabolism in cancer, non-small cell lung cancer, prostate cancer, endocrine resistance, neurotrophin signaling pathway, and breast cancer. Numerous mechanisms are recognized as significant contributors to OC, governing cell proliferation, resistance to apoptosis, metabolic reprogramming, and hormone-responsive signaling. The enhancement of the PI3K-Akt and prolactin signaling pathways indicates that medicarpin may concurrently affect cell survival, proliferation, and mechanisms of chemoresistance in OC.

The 107 common targets were mapped onto the prolactin signaling pathway using the Pathview program to elucidate mechanistic insights regarding pathway linkages (Figure 5B). The visualization indicated that multiple genes, such as PIK3CA, AKT1, MAPK1, ESR1, and SOCS3, occupy critical regulatory nodes that connect the PI3K-Akt, MAPK, and JAK-STAT pathways. These intersections are pivotal to the regulation of cell-cycle progression, DNA synthesis, apoptosis inhibition, and endocrine responsiveness, processes frequently dysregulated in OC.

The data collectively indicate that medicarpin has multi-target effects on interrelated carcinogenic pathways, especially those regulated by kinase-driven signaling and hormone-dependent transcription. The findings underscore the PI3K-Akt/mTOR and prolactin signaling pathways as prospective therapeutic targets by which medicarpin may influence OC advancement and surmount treatment resistance.

### 2.6. Validation of Principal Targets Using Molecular Docking

Molecular docking investigations were conducted to evaluate the interaction patterns of medicarpin with the 10 hub targets identified from the PPI network: CASP3, ESR1, MTOR, PIK3CA, CCND1, GSK3B, CDK4, PARP1, CHEK1, and ABL1. Table 1 summarizes the docking scores and inhibition constants for each target, indicating diverse binding affinities among the potential proteins. Of all targets, CASP3 and ESR1 demonstrated the most advantageous binding energies (−6.13 kcal/mol and −7.68 kcal/mol, respectively) together with low anticipated inhibition constants, surpassing their respective reference inhibitors (ASA for CASP3 and 53Q for ESR1). According to the comparative docking results, CASP3 and ESR1 were identified as the most potential molecular targets for mediating medicarpin’s anticancer activity in ovarian cancer, hence justifying their prioritization in further research.

**Figure 6:**
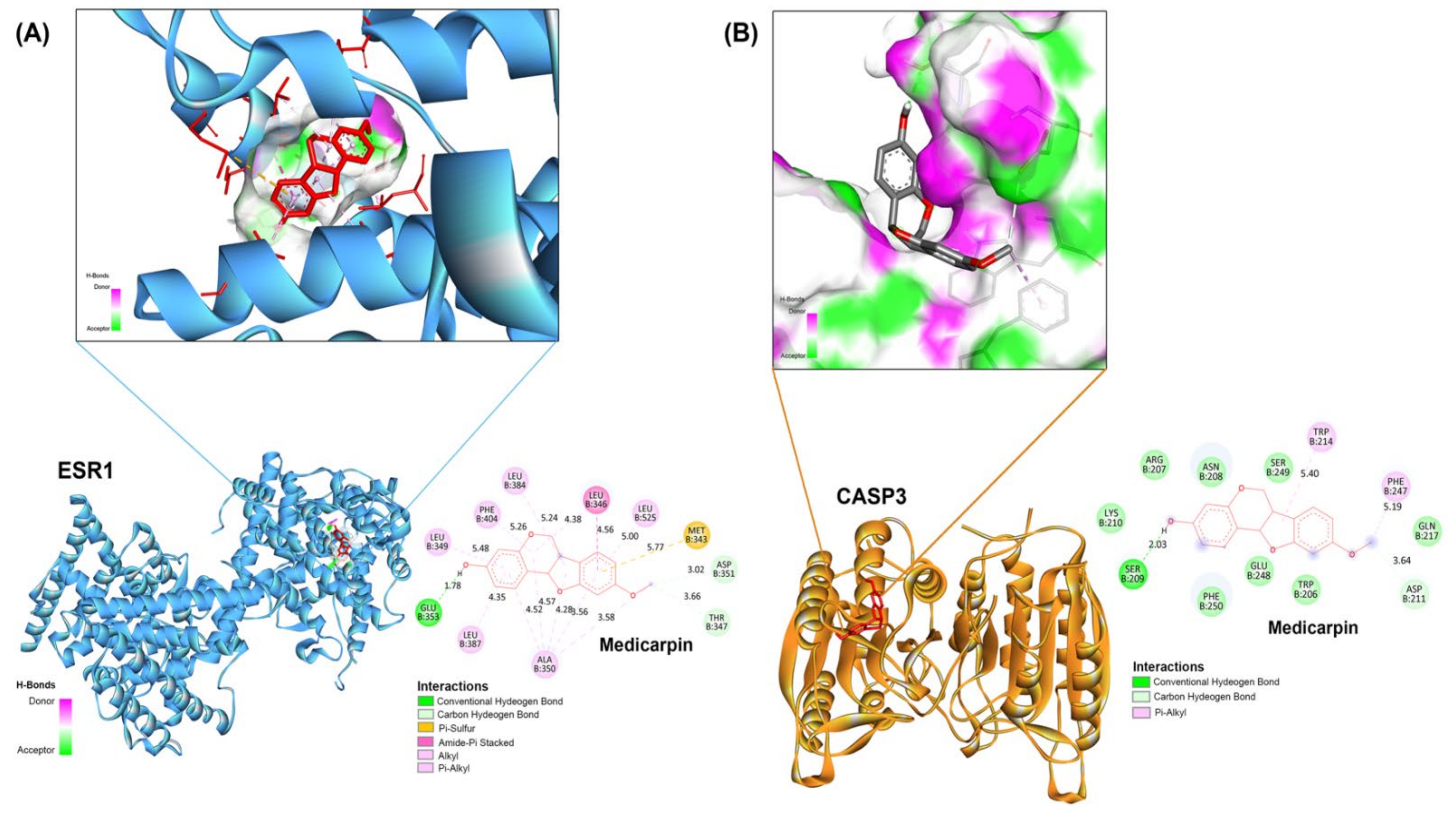
Molecular docking validation of medicarpin with ESR1 and CASP3. (**A**) Docking visualization of medicarpin bound to ESR1 (PDB: 6VPF). The inset shows the electrostatic surface of the binding pocket (green = hydrogen-bond acceptors; magenta = donors). Two-dimensional interaction mapping illustrates hydrogen bonding with GLU353 and hydrophobic contacts with LEU346, LEU387, LEU349, and PHE404, stabilizing the ligand within the receptor pocket. (**B**) Docking visualization of medicarpin bound to CASP3 (PDB: 7RN9). Medicarpin forms hydrogen bonds with SER209 and LYS210, as well as additional interactions with ARG207, ASN208, and PHE247. Insets display surface electrostatics and 2D interaction networks. These findings confirm strong and specific binding of medicarpin to ESR1 and CASP3, supporting its dual modulation of estrogen-responsive and apoptotic pathways.

In the ESR1-medicarpin complex, the ligand was securely positioned within the active pocket, establishing many hydrophobic interactions with LEU346, LEU387, LEU349, and PHE404, in addition to a hydrogen bond with GLU353 and π–alkyl contacts with MET343 and ALA350 (Figure 6A). These interactions stabilize medicarpin within the estrogen receptor binding cavity, indicating its potential to influence estrogen-responsive signaling. Medicarpin exhibited selective binding to the catalytic region of CASP3 via hydrogen bonds with SER209 and LYS210, along with supplementary interactions involving ARG207, ASN208, and PHE247 (Figure 6B). The identified binding pattern may augment the activation of apoptotic pathways by directly influencing caspase activity.

These data indicate that medicarpin exhibits a significant binding affinity for ESR1 and CASP3, underscoring its dual potential in modulating cell proliferation and death pathways in OC.

### 2.7. Prognostic Importance of CASP3 and ESR1 Expression in Ovarian Cancer

**Figure 7:**
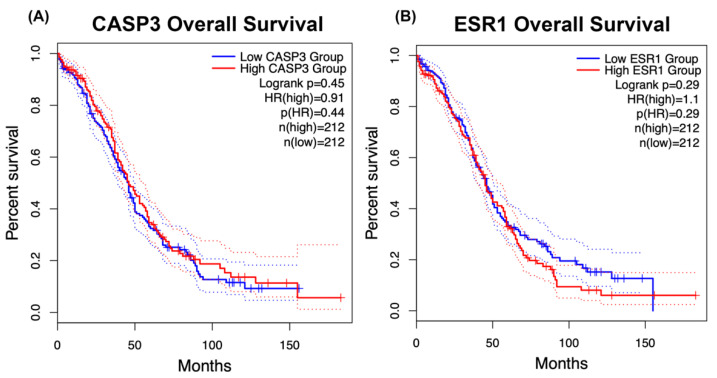
Kaplan–Meier analysis of overall survival associated with CASP3 and ESR1 expression in ovarian cancer. (**A**) Kaplan–Meier plot of overall survival based on CASP3 expression in TCGA ovarian cancer patients. The high-expression group (red) exhibits a slightly improved survival trend compared with the low-expression group (blue), although this difference is not statistically significant (log-rank *p* = 0.45; HR = 0.91). (**B**) Kaplan–Meier plot of overall survival based on ESR1 expression. The high-expression group (red) shows a minor, nonsignificant decrease in survival relative to the low-expression group (blue) (log-rank *p* = 0.29; HR = 1.10). Dotted lines indicate 95% confidence intervals. Neither gene serves as an independent prognostic marker, though both may contribute mechanistically to medicarpin’s apoptotic and hormone-responsive effects in ovarian cancer.

Kaplan–Meier survival analysis was conducted utilizing TCGA OC data to investigate the clinical significance of the discovered hub targets, specifically examining the correlation between CASP3 and ESR1 expression and overall survival. Figure 6A illustrates that patients with elevated CASP3 expression demonstrated somewhat enhanced survival relative to the low-expression cohort; yet, the difference lacked statistical significance (log-rank *p* = 0.45, HR = 0.91) (Figure 7A). Likewise, ESR1 expression had no significant correlation with overall survival (log-rank *p* = 0.29, HR = 1.10; Figure 7B). The data demonstrate that neither CASP3 nor ESR1 functions as an independent predictive marker in OC. Nonetheless, due to their permanent binding associations with medicarpin, both proteins may functionally contribute to the compound’s anticancer mechanism, particularly in regulating apoptotic and hormone-responsive pathways rather than serving as direct survival determinants. To enhance interpretability, the explanation accompanying the survival analysis has been elaborated on to more distinctly convey the clinical significance of the observed patterns. The absence of statistically significant differences between high- and low-expression groups may indicate the considerable biological variability of OC and the multifaceted factors affecting patient survival. This clarification seeks to further the knowledge of the significance of CASP3 and ESR1 expression in OC prognosis.

## 3. Discussion

This study provides an initial, thorough systems-level assessment of medicarpin as a prospective multi-target therapeutic agent in OC using integrative network pharmacology and molecular docking methodology. Through the integration of computational prediction, enrichment analysis, structural validation, and survival correlation, we elucidated the molecular framework by which medicarpin may manifest its anticancer properties. The results indicate that medicarpin primarily affects kinase-mediated and hormone-dependent signaling pathways, specifically the PI3K-Akt/mTOR and prolactin pathways, while also influencing apoptotic regulators such as CASP3. These findings establish medicarpin as a promising candidate for the development of multi-pathway therapies to address chemoresistance and disease heterogeneity in OC.

The discovery of 107 common targets between medicarpin-associated and OC-related genes highlights the compound’s ability to influence a broad range of cancer-related mechanisms. The PPI network analysis identified key oncogenic regulators, including mTOR, PIK3CA, CCND1, and ESR1, alongside pro-apoptotic molecules such as CASP3 and CHEK1, highlighting a dual role in suppressing proliferative signaling and promoting cell death. This multitarget pharmacology is especially beneficial in ovarian cancer, a disease marked by significant genomic heterogeneity and redundant survival pathways [18]. The enhancement of kinase-related Gene Ontology items, such as protein phosphorylation and ATP binding, suggests that medicarpin functions as a regulator of kinase networks. This is consistent with previous research indicating that pterocarpan derivatives inhibit oncogenic kinases, including AKT and GSK3B, resulting in cell-cycle arrest and death in breast, prostate, and liver malignancies [19,20,21].

The KEGG pathway study verified that medicarpin primarily influences the PI3K-Akt/mTOR and prolactin signaling pathways, which are crucial in OC cell proliferation, metabolic adaptability, and treatment resistance. The continual activation of the PI3K-Akt/mTOR pathway stimulates tumor proliferation and facilitates platinum-based chemoresistance by inhibiting apoptotic mechanisms and enhancing DNA repair [22,23,24]. Our observations indicate that medicarpin may inhibit these processes by targeting both upstream and downstream components, including PIK3CA, AKT1, and mTOR, therefore resensitizing tumor cells to apoptotic signals. The anticipated regulation of ESR1 and SOCS3 within the prolactin and JAK-STAT pathways suggests that medicarpin may influence hormone-responsive and cytokine-mediated transcription, both recognized factors in OC progression and endocrine resistance [22,23]. The simultaneous reduction in survival and endocrine signaling highlights medicarpin’s potential as a multi-pathway suppressor that can rebalance oncogenic signaling dynamics in OC.

Molecular docking validated these mechanistic predictions by demonstrating the favorable binding affinities of medicarpin for ESR1 (−7.68 kcal/mol) and CASP3 (−6.13 kcal/mol), exceeding those of reference inhibitors. The significant hydrogen bonding and hydrophobic contacts in ESR1’s ligand-binding domain indicate that medicarpin may operate as a partial antagonist, diminishing estrogen-mediated transcriptional activities linked to tumor growth. The ongoing attachment in CASP3’s active area suggests that it could boost caspase activation, which would enhance the natural process. This dual-target binding profile illustrates the concept of network pharmacology, wherein individual medicines attain therapeutic synergy by interacting with functionally separate yet physiologically convergent nodes [25,26].

While Kaplan–Meier survival analysis indicated that the expression of CASP3 and ESR1 alone was not significantly correlated with overall survival, the result does not undermine their mechanistic significance. Prognostic neutrality often arises from compensatory signaling and inter-patient heterogeneity in OC [27,28,29,30]. However, pharmacological reactivation or blockade of these pathways may still provide therapeutic advantages. Compounds that may co-modulate ESR1-mediated hormonal signaling and CASP3-dependent apoptosis may target two critical vulnerabilities of OC: hormone-driven proliferation and evasion of apoptosis. The findings, along with medicarpin’s advantageous ADME characteristics and adherence to Lipinski’s Rule of Five, endorse its potential repositioning as an orally bioavailable small-molecule candidate for subsequent preclinical research [31,32,33,34].

Modern OC treatments, such as PARP inhibitors, PI3K/mTOR inhibitors, and anti-angiogenic agents, target specific signaling pathways and often lead to adaptive resistance [35,36,37]. Medicarpin’s anticipated multitarget interaction with the PI3K-Akt/mTOR, MAPK, and endocrine pathways may enhance the efficacy of these medications or function as an adjunct therapy. Medicarpin’s suppression of PIK3CA and mTOR resembles the mechanisms of alpelisib and everolimus but may mitigate toxicity through partial and temporary regulation [13,20,38]. Furthermore, its association with ESR1 indicates possible compatibility with hormonal treatments or immune checkpoint inhibitors that may benefit from increased tumor immunogenicity resulting from apoptotic priming [30,39].

This research is primarily computational and generates hypotheses; in vitro and in vivo validation will be crucial to verify medicarpin’s molecular targets and subsequent consequences. An experimental evaluation of medicarpin’s ability to influence phosphorylation cascades, mitochondrial apoptosis, and estrogen receptor transcription would elucidate its mechanism. Furthermore, integrative omics encompassing transcriptomic, phosphoproteomic, and metabolomic profiling may elucidate medicarpin’s systemic influence on tumor signaling networks and metabolism [30,40,41]. Assessing medicarpin in platinum-resistant OC cell lines or patient-derived organoids will be essential to determine translational significance and therapeutic opportunities.

This integrated study clarifies the molecular framework by which medicarpin may demonstrate anticancer efficacy in OC. Medicarpin has a remarkable molecular plasticity by concurrently targeting kinase-driven oncogenic networks and hormone-dependent transcriptional circuits, unlike single-target therapies. The integration of network pharmacology, pathway enrichment, and molecular docking presents strong evidence that medicarpin may function as a lead chemical for developing multitarget therapies to address chemoresistance and clinical heterogeneity in OC. These findings establish a conceptual basis for future experimental validation and strategic medication development utilizing medicarpin’s multifaceted anticancer properties.

## 4. Materials and Methods

### 4.1. Chemoinformatics, Drug Likeness, and ADME Predictions

The cheminformatics data and drug-likeness of medicarpin were assessed using the SwissADME server (http://www.swissadme.ch; accessed on 20 September 2025) [42], an online tool designed for evaluating pharmacokinetic properties, oral bioavailability, and drug-likeness. Lipinski’s Rule of Five (RO5) was employed as a screening criterion to assess the drug-likeness of these compounds for potential oral medicines in humans [43]. The metrics being assessed included molecular weight (MW), lipophilicity (logP), topological polar surface area (TPSA), the number of rotatable bonds, hydrogen bond acceptor (HBA) and hydrogen bond donor (HBD) counts, as well as water solubility. The pharmacokinetic properties and toxicity (ADMET) profile of medicarpin were systematically assessed using two computational platforms: pKCSM (https://biosig.lab.uq.edu.au/pkcsm/; accessed on 20 September 2025), which predicts absorption, distribution, metabolism, excretion, and toxicity parameters based on graph-based signatures, and Pro-Tox-III (https://tox.charite.de/protox3/; accessed on 20 September 2025), an advanced web server designed to estimate various toxicity endpoints, including LD50 values, organ-specific toxicities, and potential adverse effects. Only compounds that met the drug-likeness criteria were selected for further analysis.

### 4.2. Prediction of Target Proteins

The identification of targets for bioactive compounds derived from medicarpin was performed using the Swiss Target Prediction databases (http://www.swisstargetprediction.ch/; accessed on 20 September 2025) [44]. To accomplish this, the canonical SMILES notation of medicarpin was compiled and entered into the Swiss Target Prediction database. Subsequently, candidate targets with high likelihood ratings were selected and standardized using the UniProt database (http://www.uniprot.org/) [45].

### 4.3. Potential Targets Associated with Ovarian Cancer

We employed the Human Gene Database (GeneCards, https://www.genecards.org/) to investigate and compile the targets related to OC [46]. Using the designated search term, we obtained and organized the relevant targets. We subsequently overlaid the expected targets of the compounds generated by medicarpin with those associated with OC, resulting in the creation of a Venn diagram. This Venn diagram was generated using an online tool at (https://bioinformatics.psb.ugent.be/webtools/Venn/; accessed on 20 September 2025) [47]. It illustrates the intersection of identified targets between the treatment agents and the disease. By pinpointing the common targets inside this junction, we assembled a list of medicines derived from medicarpin that exhibit potential for the treatment of OC.

### 4.4. Gene Ontology and Kyoto Encyclopedia of Genes and Genomes Pathway Enrichment Analysis

We conducted Gene Ontology (GO) and Kyoto Encyclopedia of Genes and Genomes (KEGG) pathway enrichment analyses [48] to deepen our understanding of the importance of key target genes. The investigation employed ShinyGO 0.82 (http://bioinformatics.sdstate.edu/go/; accessed on 20 September 2025) [49], a bioinformatics tool for characterizing and annotating gene function. We utilized a significance threshold of *p* < 0.05 in the enrichment analysis. The data were precisely depicted using bubble and bar charts. GO serves as a comprehensive resource for functional genomics, providing exact definitions of gene activities, including molecular functions. Conversely, KEGG provides graphical depictions of metabolic pathways and potential signaling pathways. The research clarifies the functional roles of essential target genes and highlights significant pathways associated with the medications under investigation.

### 4.5. Construction of the Protein–Protein Interaction (PPI) Network

To examine the functional relationships between the shared targets of medicarpin and OC, a PPI network was constructed utilizing STRING v12.0 (species: Homo sapiens) with a confidence score threshold of 0.40, hence preserving medium-confidence interactions (http://string-db.org/; accessed on 20 September 2025) [50]. The resultant dataset, consisting of proteins as nodes and anticipated interactions as edges, was exported and later imported into Cytoscape v3.10.1 (https://cytoscape.org/ accessed on 20 September 2025) for visualization and topological analysis [51]. Essential network characteristics such as node degree, clustering coefficient, and betweenness centrality were measured utilizing the Network Analyser tool to delineate the overall structural organization. CytoHubba (v0.1); accessed on 20 September 2025 was utilized to rank nodes according to degree centrality for the purpose of identifying essential regulatory proteins [52]. The 10 highest-ranked proteins were identified as hub targets, indicating their potential as critical regulators for future experimental validation.

### 4.6. Molecular Docking Studies Involving Medicarpin and Hub Genes

The three-dimensional structures of medicarpin compounds (PubChem CID 336327) were acquired from the PubChem database (http://pubchem.ncbi.nlm.nih.gov/; accessed on 20 September 2025). Medicarpin is systematically designated by its IUPAC nomenclature (6aR,11aR). -3-Methoxy-9-methyl-6a,11a-dihydro-6H-benzocchromene-6,11-diol. Ligands were optimized by specifying bond orders, angles, and topologies while incorporating any missing and polar hydrogen at pH 7.4. We prepared the ligands for docking by performing energy minimization using conjugate steepest descent methods and used charge addition for ionization correction under the AMBER force field with AM1-BCC charge assignment. The optimization techniques were conducted using UCSF Chimaera version 1.17.3. We obtained the three-dimensional crystal structures of the hub target proteins from the Protein Data Bank (PDB, https://www.rcsb.org/). We eliminated water molecules and small molecule ligands from the protein structure utilizing BIOVIA Discovery Studio. Subsequently, we employed AutoDock technologies to analyze the hub goals, including processes like as hydrogenation, charge distribution, and atomic type integration. The ligand and proteins were transformed into forms suitable with Auto-Dock (PDB and PDBQT).

Molecular docking was performed using AutoDock version 4.2. The Lamarckian genetic algorithm was employed for the molecular docking experiment, conducted using AutoDock4 software. In this approach, the protein structure was considered a rigid molecule, whereas the ligand was seen as flexible. All remaining parameters employed the default values in AutoDockTools (ADT). Fifty genetic algorithm (GA) executions were performed for conformational sampling, with a population size of 200. A docking box was constructed to completely encapsulate the binding site of the receptor protein. The quantity of binding energy was utilized to assess the likelihood of an interaction between the receptor and the ligand. The optimal conformation was determined to possess minimal binding energy (kcal/mol). The interactions of natural ligands or pharmaceuticals were compared with the optimal docked conformation of medicarpin. Binding energy data from the molecular docking method was acquired using AutoDockTools 1.5.7. Ultimately, proposed protein–ligand interactions and binding modalities were analyzed and visualized using the BIOVIA Discovery Studio Visualisers software https://www.3ds.com/products/biovia/discovery-studio/visualization; accessed on 1 October 2025 (Accelrys, San Diego, CA, USA) [53].

### 4.7. Survival Analysis of CASP3 and ESR1 Expression in Ovarian Cancer Patients

We conducted a survival analysis utilizing The Cancer Genome Atlas OC cohort (TCGA-OV) to assess the predictive significance of the two hub targets identified in this investigation, *CASP3* and *ESR1*, concerning medicarpin’s anticipated mechanism of action. Gene expression and overall survival (OS) data were sourced from the Gene Expression Profiling Interactive Analysis 2 platform (GEPIA2; http://gepia2.cancer-pku.cn/; accessed on 20 September 2025), a comprehensive database utilized for clinical-genomic correlation analyses [54]. OC patients were categorized into high-expression and low-expression groups for each gene, according to the median expression value. Kaplan–Meier survival curves were constructed to compare overall survival between the two groups. The prognostic effect was assessed by hazard ratios (HRs) with 95% confidence intervals (CIs), and statistical significance was evaluated using the log-rank test. This investigation allowed us to ascertain if fluctuations in *CASP3* and *ESR1* expression correlate with clinical outcomes in OC, thereby offering insight into their potential significance as prognostic biomarkers and as functional mediators of medicarpin’s anticancer efficacy.

## 5. Conclusions

This integrated study clarifies the molecular framework by which medicarpin may demonstrate anticancer efficacy in OC. Medicarpin exhibits a unique mechanistic plasticity by concurrently targeting kinase-driven oncogenic networks and hormone-dependent transcriptional circuits, a feat seldom achieved by single-target drugs. The integration of network pharmacology, route enrichment, and molecular docking presents strong evidence that medicarpin may function as a lead chemical for the development of multitarget therapies to address chemoresistance and disease heterogeneity in OC. These findings establish a conceptual basis for future experimental validation and strategic medication development utilizing medicarpin’s multifaceted anticancer properties.

## Figures and Tables

**Table 1:** Binding affinities and inhibition constants from molecular docking of medicarpin and reference inhibitors with ovarian-cancer-associated proteins.

No	Protein Name	PDB	Compound and Positive Control	Binding Energies (kcal/mol)	Inhibition Constant (nM)
1	CASP3	7RN9	Medicarpin	−6.13	32.24 uM
			ASA	−0.19	382.65 mM
2	ESR1	6VPF	Medicarpin	−7.68	2.37 uM
			53Q	−3.64	2.14 mM
3	CDK4	6P8F	Medicarpin	−7.87	1.57 uM
			PTR	−7.66	2.43 uM
4	CCND1	6P8F	Medicarpin	−7.91	1.6 uM
			PTR	−7.68	2.33 uM
5	MTOR	5OQ4	Medicarpin	−7.92	1.57 uM
			A3W	−7.59	2.73 uM
6	PIK3CA	5XGH	Medicarpin	−6.39	20.57 uM
			84U	−7.71	2.22 uM
7	PARP1	7KK4	Medicarpin	−7.43	3.61 uM
			09L	−11.52	3.62 nM
8	GSK3B	4ACC	Medicarpin	−6.8	10.45 uM
			7YG	−7.2	5.26 uM
9	CHEK1	2HXL	Medicarpin	−7.03	7.06 uM
			422	−12.07	1.43 nM
10	ABL1	4WA9	Medicarpin	−7.91	1.6 uM
			AXI	−10.13	37.54 nM

## Data Availability

The datasets produced and/or examined in this investigation are accessible from the corresponding author upon reasonable request. Publicly available third-party resources can be accessed at the following links: GeneCards (https://www.genecards.org/; accessed on 20 September 2025), SwissADME (https://www.swissadme.ch/; accessed on 20 September 2025), SwissTargetPrediction (https://www.swisstargetprediction.ch/; accessed on 20 September 2025), SEA (http://sea.bkslab.org/; accessed on 20 September 2025), ProTox-III (https://tox-new.charite.de/protox_III/; accessed on 20 September 2025), and KEGG (https://www.kegg.jp/; accessed on 20 September 2025). The reutilization of KEGG pathway imaging is authorized by Kanehisa Laboratories (permission letter provided).

## Supplementary Materials

The following supporting information can be downloaded at: https://www.mdpi.com/article/10.3390/ijms27010174/s1.

## Abbreviations

The following abbreviations are used in this manuscript:

ADME	Absorption, Distribution, Metabolism, and Excretion
ADMET	Absorption, Distribution, Metabolism, Excretion, and Toxicity
AhR	Aryl Hydrocarbon Receptor
AChE	Acetylcholinesterase
Akt	Protein Kinase B
AMPAR	α-Amino-3-hydroxy-5-methyl-4-isoxazolepropionate Receptor
ATAD5	ATPase Family AAA Domain-Containing Protein 5
BP	Biological Process
CASP3	Caspase-3
CCND1	Cyclin D1
CDK	Cyclin-Dependent Kinase
CDK2	Cyclin-Dependent Kinase 2
CDK4	Cyclin-Dependent Kinase 4
CYP	Cytochrome P450
Egan	Egan’s drug-likeness rule
EGFR	Epidermal Growth Factor Receptor
ER	Estrogen Receptor
ER-α	Estrogen Receptor Alpha
ER-LBD	Estrogen Receptor Ligand-Binding Domain
ESR1	Estrogen Receptor 1
FDR	False Discovery Rate
GABAR	Gamma-Aminobutyric Acid Receptor
GEPIA2	Gene Expression Profiling Interactive Analysis, version 2
GO	Gene Ontology
GSK3B	Glycogen Synthase Kinase 3 Beta
HSE	Heat Shock Factor Response Element
JAK-STAT	Janus Kinase–Signal Transducer and Activator of Transcription
KEGG	Kyoto Encyclopedia of Genes and Genomes
LD50	Lethal Dose, 50%
LOAEL	Lowest Observed Adverse Effect Level
MAPK	Mitogen-Activated Protein Kinase
mTOR	Mechanistic Target of Rapamycin
NF-κB	Nuclear Factor Kappa-light-chain-enhancer of Activated B Cells
OC	Ovarian Cancer
PAINS	Pan-Assay Interference Compounds
PARP1	Poly(ADP-Ribose) Polymerase 1
PBK	PDZ-Binding Kinase
PDB	Protein Data Bank
PI3K	Phosphatidylinositol 3-Kinase
PIK3CA	Phosphatidylinositol-4,5-Bisphosphate 3-Kinase Catalytic Subunit Alpha
PKCSM	Pharmacokinetics and Toxicity Prediction Model
PPI	Protein–Protein Interaction
ProTox-III	Prediction of Toxicity, version 3
PXR	Pregnane X Receptor
RYR	Ryanodine Receptor
SEA	Similarity Ensemble Approach
SOCS3	Suppressor of Cytokine Signaling 3
TCGA	The Cancer Genome Atlas
TPSA	Topological Polar Surface Area
TRAIL	TNF-Related Apoptosis-Inducing Ligand
